# Prognostic Role of Optic Nerve Sheath Diameter for Neurological Outcomes in Post-Cardiac Arrest Patients: A Systematic Review and Meta-Analysis

**DOI:** 10.1155/2020/5219367

**Published:** 2020-12-23

**Authors:** Yan Wei Zhang, Sheng Zhang, Hui Gao, Chao Li, Ming Xi Zhang

**Affiliations:** Emergency Department, Xingtai People's Hospital, No. 16 Hongxing Road, Qiaodong Qu, Xingtai 054031, China

## Abstract

**Objective:**

The present study investigated whether optic nerve sheath diameter (ONSD) could be used to predict neurological outcomes in post-cardiac arrest (CA) patients.

**Methods:**

We performed a comprehensive literature search in the Cochrane Library, ScienceDirect, PubMed, and Web of Science from inception to June 2020 for eligible articles. Stata 14.0 software was used to calculate the pooled odds ratios (ORs) and 95% confidence intervals (95% CIs), sensitivity, specificity, summary receiver operating characteristic (SROC) curve, subgroup analysis, sensitivity analysis, and publication bias.

**Results:**

Eight studies involving 473 patients were considered eligible for this meta-analysis. The pooled result using a random-effects model showed that broadened ONSD is associated with poor neurological outcomes in post-CA patients (OR = 15.62, 95% CI: 5.50–44.34, *P* < 0.001; *I*^2^ = 58.4%, *P* = 0.018), with a sensibility of 0.60 (95% CI: 0.45–0.73) and specificity of 0.94 (95% CI: 0.83–0.98). The area under the curve of the SROC curve for ONSD was 0.87 (95% CI: 0.84–0.90). Subgroup analysis revealed that sample size and time of ONSD measurement may be the source of heterogeneity. Sensitivity analysis demonstrated the stability of the results of this meta-analysis. No publication bias using Deeks' funnel plot was noted across the studies (*P* = 0.23).

**Conclusion:**

This meta-analysis confirmed that ONSD can be used to predict neurological outcomes in post-CA patients.

## 1. Introduction

Cardiac arrest (CA) is a health problem worldwide and is associated with high rates of mortality and morbidity. Even among those who attain return of spontaneous circulation (ROSC) after CA, in-hospital mortality remains over 50%, with a large proportion of survivors suffering permanent and severe neurological disability [1, 2]. Therefore, a valid prognostic index that can be employed early after ROSC must be developed.

Although invasive methods are considered the gold standard in intracranial pressure (ICP) monitoring, they are associated with significant risks, such as bleeding and infection [3]. The optic nerve is a part of the central nervous system and is surrounded by the dural sheath. Some reports [4–11] showed that the optic nerve sheath diameter (ONSD) is related to neurological outcomes in post-CA patients. However, the results are inconsistent. To address this discrepancy, the present study was aimed at assessing ONSD and neurological outcomes in post-CA patients.

## 2. Methods

### 2.1. Study Design

This meta-analysis was conducted on the basis of the checklists of the Preferred Reporting Items for a Systematic Review and Meta-analysis of Diagnostic Test Accuracy Studies statement [12]. All analyses were based on previous published studies; thus, no ethical approval and patient consent are required.

### 2.2. Literature Search

We performed a comprehensive literature search in the Cochrane Library, ScienceDirect, PubMed, and Web of Science from inception to June 2020 for eligible articles that assessed the association between ONSD and neurological outcomes in post-CA patients. The search strategy included a combination of the following terms: optic nerve sheath diameter AND (cardiac arrest OR resuscitation). Additionally, we searched the reference lists of selected papers and systematic reviews for potentially relevant studies missed by the original search.

All the studies have to fulfill the following criteria: (1) clinical trials assessing the diagnostic accuracy of ONSD for neurological outcomes in post-CA patients and (2) studies with complete information for the assessment of odds ratios (ORs) with 95% confidence intervals (CIs). The exclusion criteria were as follows: (1) duplicated publications or data; (2) without sufficient calculable data; and (3) commentaries, case reports, editorials, review articles, letters to the editor, expert opinions, and animal experiments.

### 2.3. Data Extraction and Quality Assessment

Two reviewers used predefined data collection forms to extract data independently. Disagreements were solved through discussions and solicitation of opinions of a third researcher. The indispensable information extracted from all primary studies included the titles, first author's name, publication year, study location, sample size, study design, cutoff value, time of ONSD measurement, method of ONSD measurement, and outcome parameters. ONSD was measured at a distance of 3 mm behind the eyeball, immediately below the sclera in a perpendicular vector in reference to the linear axis of the nerve [13].

The methodological quality of the included studies was assessed using the Revised Quality Assessment of Studies of Diagnostic Accuracy Included in Systematic Reviews- (QUADAS-) 2 tool [14]. Any disagreements were resolved by consensus, involving a third reviewer if necessary.

### 2.4. Statistical Analysis

Stata version 14.0 (StataCorp, College Station, Texas) was used in all statistical analyses. Pooled ORs and 95% CIs were used to assess the strength between ONSD and neurological outcomes in post-CA patients. The heterogeneity of studies was measured using the *I*^2^ statistic and *Q* test. *I*^2^ > 50% indicates high heterogeneity. A random-effects model was used; otherwise, a fixed-effects model was used. Deeks' funnel plot asymmetry test was performed to assess publication bias. Subgroup analysis was performed according to sample size, study design, cutoff value, time of ONSD measurement, and method of ONSD measurement for evaluating outcomes. We performed a sensitivity analysis to estimate the influence of an individual study on the recalculated OR and heterogeneity by omitting one study. A two-tailed *P* value less than 0.05 indicates statistical significance.

## 3. Results

### 3.1. Literature Search

**Figure 1:**
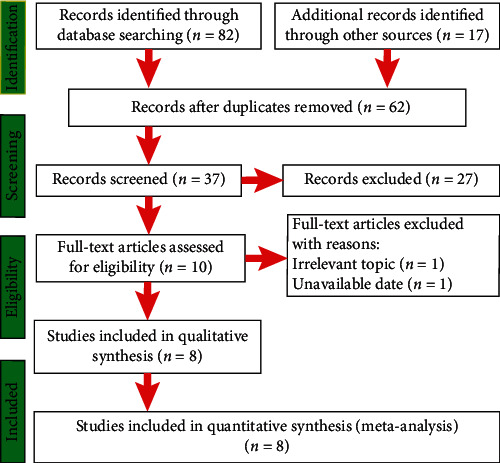
Flow chart of the study selection process.

Figure 1 presents the details regarding the screening process. A total of 99 potentially relevant records were initially identified by keyword search. Among them, 89 were excluded due to nonrelevance to the current study and/or duplication. Among 10 full studies, one was excluded for insufficient data, and one was excluded for focusing on an irrelevant topic. Finally, eight studies [4–11] were included in this meta-analysis.

### 3.2. Characteristics of the Included Studies

Table 1 shows the main characteristics of the included studies [4–11]. All these studies [4–11] were published between 2014 and 2019. Among them, six studies [4, 7–11] were based in Asia, and two [5, 6] were based in Europe. The sample sizes of the included studies ranged from 17 to 119. The cutoff values of ONSD were inconsistent across all studies, ranging from 4.90 to 6.69.

### 3.3. Quality Assessment

**Figure 2:**
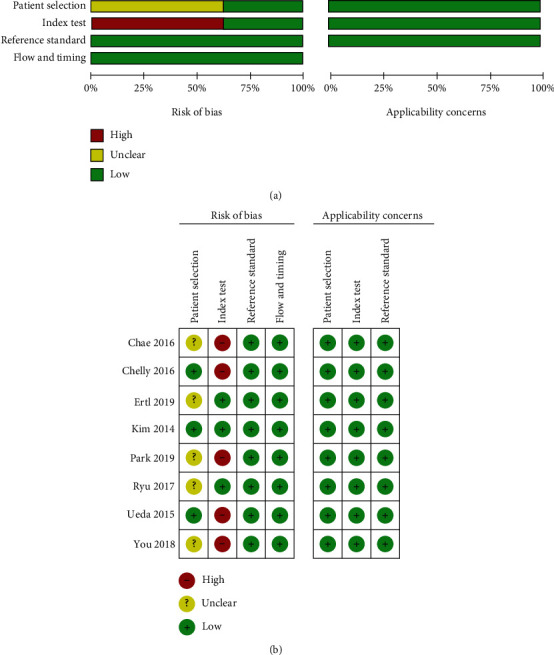
Risk of bias (a) within each study and (b) across studies.

Before data analysis and synthesis, the quality of eligible studies was evaluated using the QUADAS-2 questionnaire (Figure 2). Six studies [4, 6, 8, 9, 11] were rated as having unclear risks of overall bias due to unmentioned consecutive patients, and six studies [4, 5, 8, 10, 11] were rated as having high risks of bias in the index test due to no preset specificity.

### 3.4. ONSD and Neurological Outcomes

**Figure 3:**
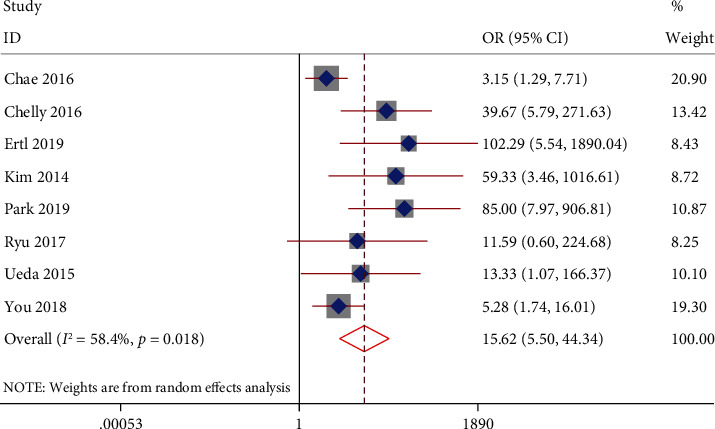
Forest plot of the association between optic nerve sheath diameter and neurological outcomes.

**Figure 4:**
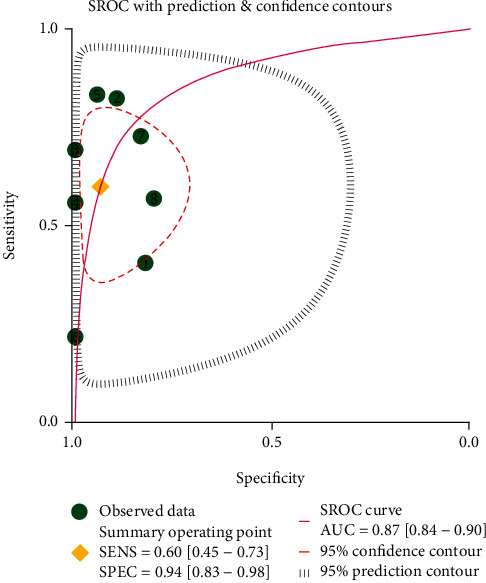
Summary receiver operating characteristic curve of the meta-analyzed sensitivity and specificity for optic nerve sheath diameter applications.

Meta-analysis using the random-effects model showed that broadened ONSD was associated with poor neurological outcomes in post-CA patients (OR = 15.62, 95% CI: 5.50–44.34, *P* < 0.001; *I*^2^ = 58.4%, *P* = 0.018; Figure 3), with a sensibility of 0.60 (95% CI: 0.45–0.73) and specificity of 0.94 (95% CI: 0.83–0.98). As shown in Figure 4, the area under the curve (AUC) of the summary receiver operating characteristic curve for ONSD was 0.87 (95% CI: 0.84–0.90).

### 3.5. Subgroup Analysis

Subgroup analysis was conducted on the basis of sample size (≤60 or >60), study design (retrospective or prospective), cutoff value (≤5.5 or >5.5 mm), time of ONSD measurement (≤24 h after ROSC or >24 h after ROSC), and method of ONSD measurement (ultrasound or computed tomography), and the results indicated that these factors did not affect the predictive value of ONSD for neurological outcomes in post-CA patients (Table 2). In addition, subgroup analysis revealed that sample size and time of ONSD measurement may be the source of heterogeneity.

### 3.6. Sensitivity Analysis

**Figure 5:**
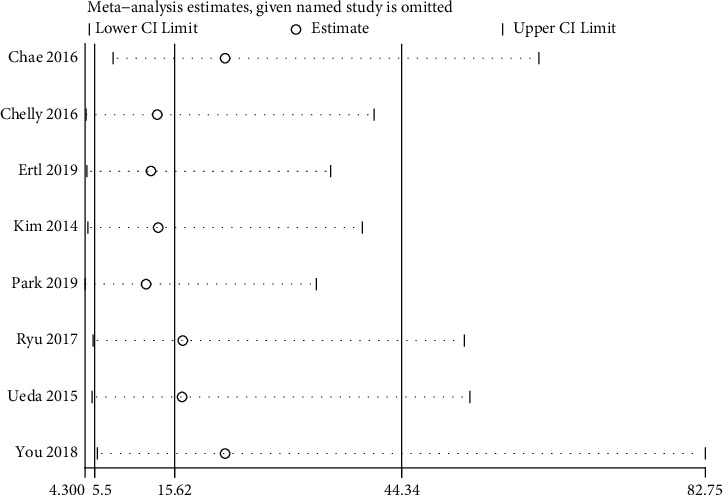
Sensitivity analysis of optic nerve sheath diameter for neurological outcomes.

As shown in Figure 5, the results of **the** sensitivity analysis did not show any significant impact from any single research study and confirmed the results of the overall performance of the ONSD.

### 3.7. Publication Bias

**Figure 6:**
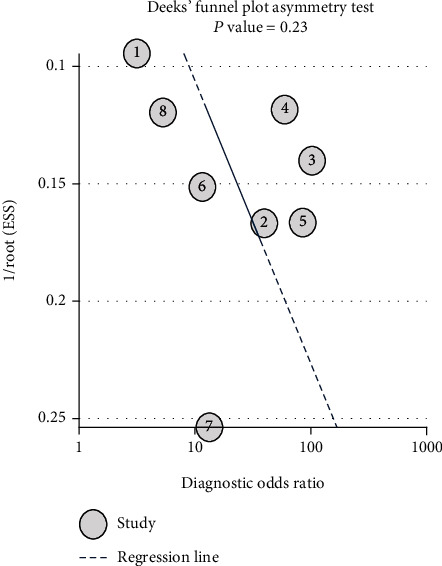
Funnel plot checking for publication bias.

As shown in Figure 6, the *P* value for Deeks' funnel plot asymmetry test was 0.23, indicating that no publication bias was observed in this meta-analysis.

## 4. Discussion

The prediction of neurological outcomes in comatose patients that were resuscitated is very important to reduce unnecessary costs, facilitate organ donation, and direct counseling with the patients' families. In this meta-analysis, we found that increased mean broadened ONSD was associated with poor neurological outcomes in post-CA patients with a sensitivity of 60%, specificity of 94%, and AUC of 0.87.

The variation in ICP is correlated with the ONSD because increased ICP is transmitted to the subarachnoid space surrounding the optic nerve, causing optic nerve sheath expansion. ONSD expansion can be compared to papilledema, but unlike papilledema, ONSD expansion occurs within seconds of an acute rise of ICP [15]. Some studies reported that a remarkable linear relationship exists between invasive ICP measurement and simultaneous ultrasonographic measurements of ONSD with a correlation coefficient of 0.42–0.78 [16–20].

However, the normative values for a normal ONSD and its associated factors remain unclear. Kim et al. [21] found that the ONSD of healthy adults was 4.11 mm with a range of 4.09–4.14 mm, whereas Bäuerle et al. [22] reported that the mean ONSD was 5.4 mm with a range of 4.3–7.6 mm. Recently, a meta-analysis included a total of 34 studies with cumulatively 2,450 patients without elevated ICP and reported that the pool of mean ONSD measurements was 4.78 mm (95% CI: 4.63–4.94) [23]. In addition, the ONSD was independently associated with eyeball transverse diameter rather than sex, body mass index, or height [21–23].

Despite relatively reliable sensitivity and specificity, a consistent ONSD cutoff to predict neurological outcomes in post-CA patients remains to be identified. Some of the included studies determined an optimal cutoff anywhere from 4.90 nm to 6.69 mm. A typical optic nerve sheath is less than 5 mm and is considered to be a good neurological outcome for post-CA patients. Future studies should establish the best cutoff values for ONSD that correlate with poor neurological outcome and that can be standardized for diverse subgroups categorized by age, temperature treatment, and condition [24].

We acknowledge several limitations to our large meta-analysis. First, our results are subject to limitations inherent to any meta-analysis on the basis of pooling of data from different studies with different inclusion criteria, different designs, variable follow-up duration, and different patient populations. Second, most studies included in the present meta-analysis were retrospective and had a relatively small sample size, with the potential for patient selection bias. Third, most studies originated from Asia, potentially limiting the generalizability to other healthcare systems.

In conclusion, our findings demonstrate that ONSD is a valuable and noninvasive marker to predict neurological outcomes in post-CA patients.

## Figures and Tables

**Table 1:** Characteristics of the included studies.

Author (year)	Publication year	Country	Design	Method of ONSD measurement	Time of ONSD measurement	Sample size	Cutoff (mm)	ONSD	
								Poor prognosis	Good prognosis
Chae et al. [4]	2016	Korea	Retrospective	CT	Within 6 h after ROSC	119	5.9	5.8 (0.6)	5.6 (0.5)
Chelly et al. [5]	2016	France	Prospective	Ultrasound	At 24 h after CA	36	5.5	7.2 (6.8–7.4)	6.5 (6.0–6.8)
Ertl et al. [6]	2019	Germany	Prospective	Ultrasound	At 24 h after CA	49	5.75	5.88 ± 0.44	5.36 ± 0.43
Kim et al. [7]	2014	Korea	Retrospective	CT	Within 24 h after ROSC	91	6.21	6.29 ± 0.46	5.57 ± 0.30
Park et al. [8]	2019	Korea	Prospective	Ultrasound	At 24 h after ROSC	36	4.90	5.24 ± 0.56	3.78 ± 0.83
Ryu et al. [9]	2017	Korea	Retrospective	CT	Within 24 h after ECPR	42	6.69	6.07 (5.71–6.64)	5.57 (5.14–5.98)
Ueda et al. [10]	2015	Japan	Retrospective	Ultrasound	At 12–72 h after CA	17	5.4	6.1 (5.4–7.2)	5.0 (4.4–6.1)
You et al. [11]	2018	Korea	Retrospective	Ultrasound	Before TTM	83	5.11	5.29 (4.50–5.76)	4.48 (4.27–5.09)

CA: cardiac arrest; ECPR: extracorporeal cardiopulmonary resuscitation; NA: not available; ONSD: optic nerve sheath diameter; ROSC: return of spontaneous circulation; TTM: target temperature management.

**Table 2:** Subgroup analysis.

	*N*	OR (95% CI)	*P*	*I* ^2^ (*P* value)
Sample size				
<60	5	36.76 (12.32–109.66)	<0.001	0.0% (0.706)
≥60	3	5.71 (1.80–18.13)	0.003	54.7% (0.110)
Study design				
Retrospective	5	5.95 (2.58–13.71)	<0.001	24.8% (0.256)
Prospective	3	61.40 (16.26–231.93)	<0.001	0.0% (0.818)
Cutoff value				
≤5.5 mm	4	17.86 (4.59–69.54)	<0.001	51.8% (0.101)
>5.5 mm	4	16.61 (2.18–126.74)	0.007	68.5% (0.023)
Time of ONSD measurement				
≤24 h after ROSC	4	5.78 (2.18–15.28)	<0.001	37.4% (0.188)
>24 h after ROSC	4	44.08 (13.60–142.86)	<0.001	0.0% (0.680)
Method of ONSD measurement				
Computed tomography	3	9.25 (1.28–66.70)	<0.001	59.6% (0.084)
Ultrasound	5	22.83 (6.22–83.88)	<0.001	51.2% (0.085)

CI: confidence intervals; OR: odd ratio; ROSC: return of spontaneous circulation.

## Data Availability

The data used to support the findings of this study are available from the corresponding author upon request.
